# NKG2C^+^ memory-like NK cells contribute to the control of HIV viremia during primary infection: Optiprim-ANRS 147

**DOI:** 10.1038/cti.2017.22

**Published:** 2017-07-07

**Authors:** Françoise Gondois-Rey, Antoine Chéret, Samuel Granjeaud, Françoise Mallet, Ghislain Bidaut, Camille Lécuroux, Mickaël Ploquin, Michaela Müller-Trutwin, Christine Rouzioux, Véronique Avettand-Fenoël, Alessandro Moretta, Gilles Pialoux, Cécile Goujard, Laurence Meyer, Daniel Olive

**Affiliations:** 1 Aix Marseille University, CNRS, Inserm, Institut Paoli-Calmettes, CRCM, Department of Immunity and Cancer, Marseille, France; 2 Department of Internal Medecine, Hôpital Bicêtre, APHP, Le Kremlin-Bicêtre, France; 3 EA 7327 Paris Descartes University, Paris, France; 4 Aix Marseille University, CNRS, Inserm, Institut Paoli-Calmettes, CRCM, CiBi Platform, Marseille, France; 5 Paris-Sud University, U1184, Le Kremlin-Bicêtre, France; 6 CEA, Department of DSV/iMETI, IDMIT, Fontenay-aux-Roses, France; 7 Inserm U1184, Department of ImVA ‘Immunology of chronic Viral infections and Autoimmune diseases’, Le Kremlin-Bicêtre, France; 8 Institut Pasteur, HIV, Inflammation and Persistance Unit, Virology Department, Paris, France; 9 Virology Laboratory, APHP CHU Necker-Enfants Malades, Paris, France; 10 Dipartimento di medecina Sperimentale and Centro di Eccellenza per la Ricerca Biomedica, Università di Genova, Genova, Italy; 11 Department of Infectious Diseases APHP, Hôpital Tenon, Paris, France; 12 Inserm, CESP U1018, Univ Paris-Sud, Department of Epidemiology and Population Health, APHP, Hôpital Bicêtre, Le Kremlin-Bicêtre, France

## Abstract

Natural-killer (NK) cells are important immune effectors during a viral infection. Latent CMV infection is widely spread and was demonstrated to shape the NK cell repertoire through the NKG2C receptor. An expansion of NKG2C^+^ NK cells has been reported during primary HIV infection (PHI), but their role is not known. We previously found a correlation between the maturation state of the NK cell compartment and a lower viral load by studying patients from the ANRS 147 Optiprim trial. We investigated here extensively the NKG2C^+^ NK cells at the time of PHI and its evolution after 3 months of early antiretroviral therapy (combination antiretroviral therapy (cART)). Multiparametric cytometry combined with bioinformatics was used to determine subsets. NK^bright^ NKG2C^+^ progenitor, NK^dim^ NKG2C^+^ effector and NK^dim^ NKG2C^+^CD57^+^ memory-like populations were identified. Two groups of patients were unraveled according to the distribution of the NKG2C^+^ subsets skewed toward either progenitor/effector or memory-like phenotype. Patients with high NKG2C^+^CD57^+^ NK cell frequencies showed lower HIV-RNA, lower immune activation, higher pDC counts and reached more rapidly undetectable levels of HIV-RNA at M1 under cART. NKG2C^+^CD57^+^ NK cell frequency was the only factor strongly correlated to low viral load among other clinical features. While the patients were cytomegalovirus (CMV) infected, there was no sign of reactivation of CMV during PHI suggesting that memory-like NK cells were already present at the time of HIV infection and constituted a preexisting immune response able to contribute to natural control of HIV. This parameter appears to be a good candidate in the search of predictive markers to monitor HIV remission.

Natural-killer (NK) cells are one of the major innate immune components responsible for the rapid response of the host to invading virus.^1^ Their activity during the acute phase of viral infections can impact the quality of the adaptive immune response and the overall outcome of the infection.^2^ Because of their pivotal position in immune response, they are at the center of interest of many human disease studies. NK cell activity is regulated through activating and inhibitory receptors. In addition, their function is intrinsically linked to their maturation.^3^ Thus, the NK cell compartment comprises variegated subsets according to maturation, receptors expressed and functional potential, constituting a unique individual NK cell repertoire in each individual. The important role of the NK cell repertoire was recently demonstrated by the correlation between a highly diverse repertoire and risk of HIV infection.^4^

CMV is a widespread latent infection affecting 50% of people in the childhood and was reported as an important factor shaping the NK cell repertoire.^5^ CMV is the only virus known to drive the expansion of a specific NK cell subset, characterized by upregulation of the activating NKG2C receptor. Stabilization of HLA-E, the only cognate ligand of the NKG2C/CD94 dimer, by CMV-UL40 viral peptides in infected cells is the mechanism involved.^6^ During CMV reactivation, NKG2C^+^ NK cells expand temporally, then contract into mature CD57^+^ NK cells once viremia is controlled.^5, 7, 8^ NKG2C^+^CD57^+^ NK cells persisting after CMV infection are now considered as adaptive or memory-like form of NK cells.^8, 9, 10^ They express KIR receptors specific for self-HLA class I molecules and are prone to cytokine secretion after activation.^8^ Recently, adaptive NK cells have been characterized by enhanced potential for broad antiviral responses.^11^ In CMV-infected asymptomatic people, the magnitude of memory NKG2C^+^ NK cell frequencies is variable. Individual factors, including the relative contribution of NK- and T-cell-mediated control of CMV infection, or genetic factors (a homozygous deletion of the *NKG2C* gene was reported^12^) influence this variability.

Because of the prevalence of CMV infection, any new infection has a high probability to occur in the context of a CMV co-infection, and because CMV shapes the NK cell repertoire through the NKG2C receptor, NKG2C^+^ NK cell constitute an interesting subset to follow. Expansion of NKG2C^+^ NK cells was described in response to infections with hantavirus,^13^ chikungunya,^14^ hepatitis C virus (HCV)^15^ and HIV^16, 17, 18^ in the context of underlying CMV infection. NKG2C^+^ efficiently contributed to control hantavirus and chikungunya infection.^13, 14^ However, if NKG2C^+^ NK cells in HIV infection play a role on viral control remains so far unanswered.

In HIV-infected individuals, impairment of NK cell functions are detected already at the time of primary HIV infection (PHI) concurrently with a decrease of immature CD56^bright^ (NK^bright^) and increase of CD56^dim^ (NK^dim^) cell frequencies.^19^ A functionally compromised CD56^−^ NK cell subset expands later in chronically HIV-infected untreated patients.^20^ Increase of NKG2C^+^ and decrease of NKG2A^+^ NK cell populations were described in primary HIV-infected patients,^16, 18^ and an overall increase of CD57^+^ NK cells was observed in chronically HIV-infected patients.^21^ The hypothesis of interplays between HIV and NKG2C^+^ NK cells was further supported by the observation of an increased risk for HIV infection of patients carrying a NKG2C gene deletion.^22^ However, the contribution of the NKG2C^+^ NK cells to the control of HIV viremia is not well understood.

The Optiprim trial was designed to evaluate if an intensive antiviral therapy starting during PHI contributes more efficiently to a decrease of the HIV reservoir size and helps to achieve a so-called post-treatment controller status.^23^ Among the parameters potentially involved in the equilibrium between host and virus, innate immune factors were investigated in a subgroup of 30 patients. We previously found a correlation between an increase of mature NK cells, lower HIV viral load at inclusion and early response to combination antiretroviral therapy (cART).^24^ We investigated here more deeply the NKG2C^+^ NK cell compartment at inclusion and 3 months after cART onset in PHI, in relation with the CMV status of the patients. NKG2C^+^ expansion was analyzed by multiparametric cytometry including markers known to identify NK cells maturation. Since NK cell subpopulations expressing unexpected combinations of markers might be expanded in pathological conditions, bioinformatics was used to analyze patient’s NKG2C^+^ cell compartments in an unbiased approach. This allowed us to explore the contribution of NKG2C^+^ NK cell subsets to viral dynamics during PHI and after early cART.

## Results

### The high levels of NKG2C^+^ NK cells found in primary HIV-infected patients persisted after early cART

**Figure 1:**
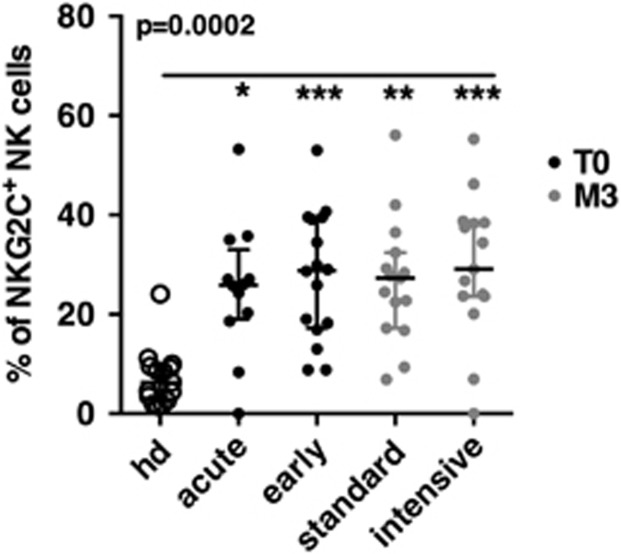
NKG2C^+^ NK cells expansion in HIV-primary infected patients. Percentage of NKG2C^+^ within NK cells in 15 healthy blood donors (HD) (open circles) and in the 30 primary HIV-infected patients at inclusion (T0) in groups of acutely or early infected (black dots), and after 3 months of cART (M3) in groups of standard or intensive cART regimens (gray dots). *P*-values from Kruskall–Wallis test is indicated on top of the groups, *P*-values from Dunn’s multiple comparison post-test on top of the pairs: *<0.05; **<0.01; ***<0.001.

NKG2C^+^ NK cells were studied in 30 patients from the ANRS 147 Optiprim clinical trial.^23^ Fourteen patients presented at a very early stage of PHI, named here ‘acute’ PHI, and defined by one or no band on the HIV-1 western blot, while 16 patients presented at a later stage of PHI, named here ‘early’ PHI and defined by the detection of more than one band (Table 1). The estimated time between infection and enrollment were, respectively, of 34 (20–53) and 37 (22–55) days. In the 15 patients treated with the standard cART regimen, 3 were acutely infected and 12 were early infected at enrollment. In the 15 patients treated with the intensive cART regimen, 11 were acutely infected and 4 were early infected at enrollment. NK cells were studied at the point of inclusion (T0) and after 3 months of cART. We first evaluated the overall percentages of NKG2C^+^ among total NK cells. When compared to healthy blood donors, we observed significantly higher levels of NKG2C^+^ NK cells in patients with PHI, irrespectively of the PHI stage at enrollment (Figure 1). These high levels persisted after 3 months of cART, independently of the cART regimen and remained highly variable (Figure 1).

### NKG2C^+^ NK cells included several subsets whose frequencies segregate two groups of patients during PHI

To gain an insight into the role of NKG2C^+^ NK cells, we analyzed their phenotype of maturation. In the NK maturation process, NK^bright^ (CD56^bright^CD16^−^NKG2A^+^) are progenitors of NK^dim^ (CD56^dim^CD16^+^), the latter being endowed with cytotoxic functions.^3^ NK^dim^ sequentially mature into subpopulations characterized by the loss of NKG2A, while CD57, a marker of senescence, is acquired on fully matured NK cells.^25^ In addition to NK cells with immature (NK^bright^) and mature phenotype (NK^dim^), a third group of NK cells (NK^low^) considered as dysfunctional, has been described in HIV-infected patients.^20^ Using CD56, CD16, NKG2A and CD57 co-expression, we sought to search for the distinct subsets within the pool of NKG2C^+^ NK cells. Because NK cells segregate into various cell subpopulations and population gating is improved by the simultaneous consideration of all markers, we used multiparametric cytometry combined with a bioinformatics analysis process, according to a previously validated method, allowing an unbiased definition of subsets^26^ (Supplementary Figure S3). Among the 10 populations identified, two showed a typical signature of immature NK cells (CD56^bright^CD16^−^NKG2A^+^, CD56^bright^CD16^+^NKG2A^+^), five displayed different levels of maturity (CD56^dim^CD16^+^NKG2A^+^CD57^−^; CD56^dim^CD16^+^NKG2A^−^CD57^−^; CD56^dim^CD16^+^NKG2A^+^CD57^+^; CD56^dim^CD16^+^NKG2A^−^CD57^+^; CD56^dim^CD16^low^NKG2A^−^CD57^+^) and three showed dysfunctional phenotypes (CD56^low^NKG2A^+^; CD56^low^CD16^low^; CD56^neg^CD57^+^CD16^+^). Thus, by homology with known phenotype markers of NK cell maturation, multiparametric cytometry combined with bioinformatics reveals that the NKG2C^+^ NK cells expansion includes 10 variegated subsets of NK cells each displaying a distinct stage of maturation.

**Figure 2:**
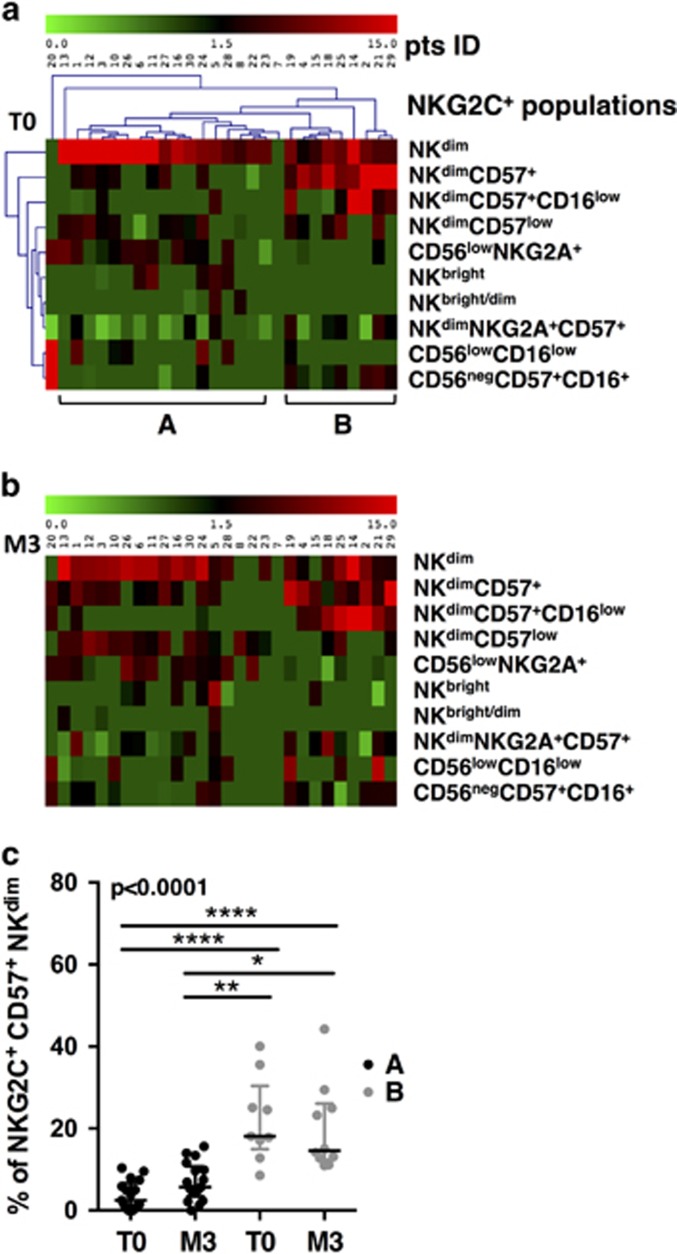
Patients grouping according to distribution of NKG2C^+^ NK cells. (**a**) Double clustering of the 10 NKG2C^+^ phenotypes shown on right (lines) according to their frequencies among NK cells of patients (columns) on a MeV heatmap. The squares display the respective frequencies according to the color scale shown on top. (**b**) Unclustered map of the frequencies of the 10 NKG2C^+^ phenotypes in patients at M3. Patients are listed in the same order as in **a**. (**c**) Evolution of the frequency of NKG2C^+^CD57^+^ subsets for patient groups A and B, at T0 and at M3. *P*-values from Kruskall–Wallis test is indicated on top of the groups, *P*-values from Dunn’s multiple comparison post-test on top of the pairs: *<0.05; **<0.01; ***<0.001.

We investigated the individual distribution of the 10 NKG2C^+^ NK cell subsets during PHI. Frequencies within the total NK cells were calculated, represented on a MeV heatmap and the HCL tool of Mev was used to cluster patients and populations (Supplementary Table S1,Figures 2a and b). At T0, three populations were the most frequent: NKG2C^+^NK^dim^, NKG2C^+^NK^dim^CD57^+^ and NKG2C^+^NK^dim^CD57^+^CD16^low^. The seven remaining populations were either rarely found or present at low frequencies. Two main groups of patients appeared based on, respectively, high percentages of NKG2C^+^NK^dim^ (group A) or high percentages of CD57^+^ subsets (NKG2C^+^NK^dim^CD57^+^ or NKG2C^+^NK^dim^CD57^+^CD16^low^) (group B). The ratios of the NKG2C^+^NK^dim^/NKG2C^+^CD57^+^ frequencies were significantly different between A and B (Supplementary Figure S5A). Two patients showed a unique profile: patient no. 7 had no NKG2C^+^ and patient no. 20 had only NKG2C^+^ NK cells corresponding to phenotypically dysfunctional populations (CD56^low/−^ and CD16^low^). Thus, the analysis of the NKG2C^+^ expansion into maturation subsets allows forming groups of patients significantly different according to a NKG2C^+^ profile skewed either to NK^dim^ CD57^−^ or to NK^dim^ CD57^+^ subsets.

### The individual frequencies of the mature NKG2C^+^ NK cells do not change after early cART

We analyzed the distribution of the subsets in the same patients at M3, using the same method, to search for individual modifications undergone during the 3 months of cART (Figure 2b). M3 frequencies revealed to be very close to that of T0. No significant modifications regarding the distribution of NKG2C^+^ subsets could be seen after 3 months of cART within the same group of patients (Supplementary Figure S5B). While there was a trend of the mature NK subsets (NK^dim^CD57^+^) to increase at M3 in group A and to decrease in group B (Supplementary Figure S5B), they were still lower in group A than in group B at M3 (Figure 2c). Altogether, the distribution of NKG2C^+^ NK cells after 3 months of early cART were variable between individual patients, but not different from that at PHI before cART onset.

### The high frequencies of NKG2C^+^ NK cell were independent of CMV reactivation during PHI

**Figure 3:**
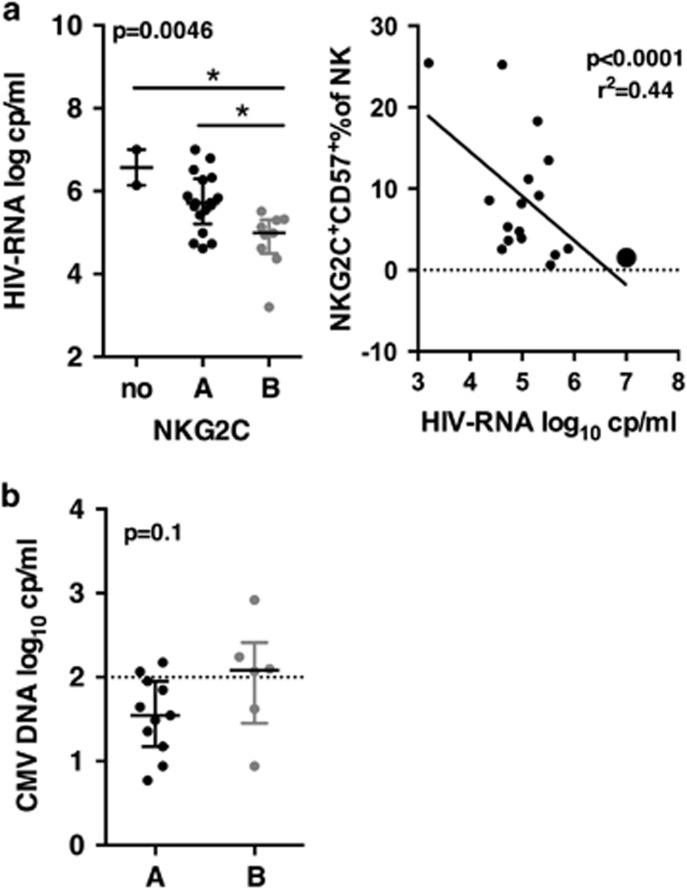
HIV and CMV viremia at T0. (**a**) Left, HIV viremia of groups A and B, and of patients without functional NKG2C. *P*-values from Kruskall–Wallis test is indicated on top of the groups, *P*-values from Dunn’s multiple comparison post-test on top of the pairs: *<0.05; **<0.01; ***<0.001. Right, correlation of percentages of NKG2C^+^NK^dim^CD57^+^ with HIV-RNA. Pearson correlation and *P*-values derived from a linear regression. Patient B who became later a post-treatment controller is indicated by a large dot. (**b**) CMV viremia. *P*-value derived from a Mann–Whitney test is indicated.

CMV is known to drive the expansion of NKG2C^+^ NK cells. A higher prevalence of CMV infection was found in populations at risk for HIV infection^27^ and CMV reactivation was frequently observed in chronically infected untreated patients, as a consequence of HIV immune suppression.^28^ In our study, 29 patients were IgG seropositive for CMV and one seronegative patient showed detectable levels of CMV-DNA suggesting a recent infection. The levels of CMV-IgG were not different between patients from groups A and B (not shown). IgM was found in one patient from each group in the presence of IgG. A trend for higher levels of CMV-DNA was observed in patients B as compared to A (median of 2.08 vs 1.54 CMV-DNA log_10_ copies per ml; *P*-value=0.1) (Figure 3b). However, in both groups, CMV-DNA levels were below or close to the threshold usually indicative of reactivation (1.9 log_10_ CMV-DNA copies per ml±0.6). Thus, while most patients were co-infected by CMV, there was no indication for a reactivation of CMV during PHI. Therefore, reactivation of CMV is unlikely to explain the higher levels of mature NKG2C^+^ during PHI in these patients.

### NK^dim^CD57^+^NKG2C^+^ NK cells frequency is inversely correlated to HIV viral load during PHI

To evaluate the impact of NKG2C^+^ expansion on HIV infection before treatment onset, we compared clinical and virological parameters of the patients at T0. Age, timing after infection, phase of the primary infection (acute or early), CD4 and CD8 counts, and HIV-DNA at T0 were not different between groups A and B, while the two patients with no NKG2C^+^ NK cells showed a trend to lower CD4 counts and higher HIV-DNA (Table 2, Supplementary Figure S6). HIV-RNA was the only significant difference between A and B: patients from group A displayed higher mean of HIV-RNA than patients from group B (5.72 log_10_ HIV-RNA copies per ml for A vs 4.77 for B), while the two patients lacking potentially functional NKG2C^+^ populations displayed even higher HIV viremia than patients from group A (mean of 6.57 log_10_ RNA copies per ml) (Figure 3a).

Then, we searched which NKG2C subsets were accounting for the striking difference of viral load between A and B. Correlations between HIV viremia and frequencies of NKG2C^+^ subsets were assessed. Positive correlations were found for NK^bright^ and CD56^low^NKG2A^+^ subsets (*P*-value of respectively 0.019 and 0.04; *r*^2^ 0.19 and 0.15). Negative correlations were found for NK^dim^NKG2C^+^CD57^+^ and NK^dim^NKG2C^+^CD57^+^CD16^low^ subsets (*P*-value of resp. 0.0001 and 0.02; *r*^2^ of resp. 0.44 and 0.19) (Figure 3b). Accordingly, the most important NKG2C^+^ subsets involved in the lower HIV viral load were those present in patients of group B, that is, NK^dim^CD57^+^ subsets.

Other parameters such as time between infection and inclusion, age and primary infection status might also influence viral load at inclusion and were indeed different for each group. To determine whether these parameters were confounders, multivariate statistical analysis including all these parameters was applied to explain HIV viral load. Only the frequency of NKG2C^+^CD57^+^ at inclusion was inversely correlated (*P*-value=0.0006; adjusted *r*^2^=0.42). Accordingly, the frequency of NKG2C^+^CD57^+^ was the only parameter among those examined strongly correlated to a lower viral load at inclusion.

### Patients with high frequencies of memory-like NK cells displayed better immunological parameters

**Figure 4:**
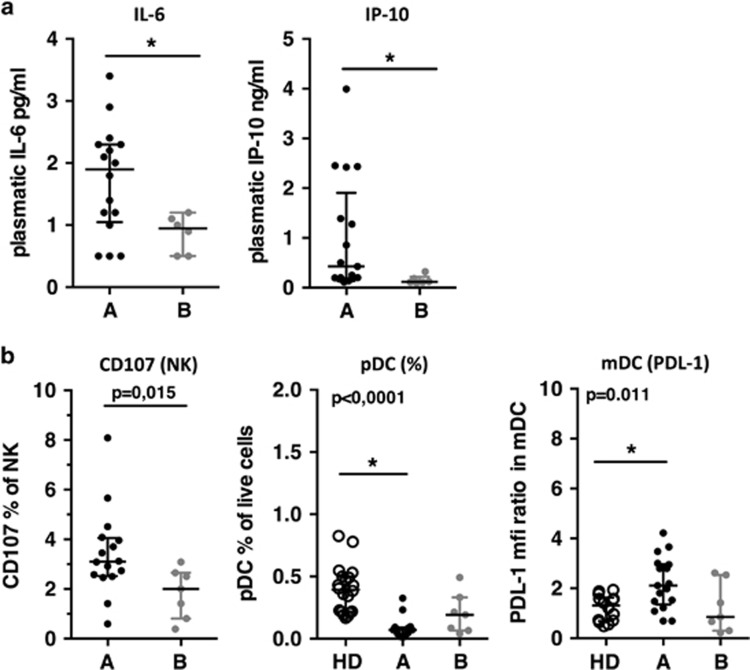
Immune status of patient groups at T0. (**a**) Left to right, plasma levels of IL-6 and IP-10. *P*-values are derived from a Mann–Whitney test. (**b**) Left to right, spontaneous degranulation of NK cells, frequencies of plasmacytoid dendritic cells (pDC) and PDL-1 expression on myeloid dendritic cells (mDC), for patient of groups A and B and a group of 15 healthy donors (HD). *P*-values from a Kruskall–Wallis test is indicated on top of the groups, *P*-values from Dunn’s multiple comparison post-test on top of the pairs: *<0.05; **<0.01; ***<0.001.

We next addressed the question whether patients of group A and B were different with respect to other major disease progression markers. Plasmatic inflammatory markers (interleukin (IL)-6, interferon γ inducible protein (IP))^29, 30^ and markers of innate immune activation and exhaustion, including plasmacytoid dendritic cells (pDC) frequency,^31^ NK spontaneous degranulation^32^ and PDL-1 expression on myeloid populations^33^ were assessed. Patients of group A displayed higher IL-6 and IP-10 levels (Figure 4a), higher expression of PDL-1 on mDC, higher frequencies of degranulating NK cells and lower pDC frequencies in blood as compared to patients from group B (Figure 4b). Patients from group B showed levels of PDL-1 on mDC and frequencies of pDC similar to those of healthy donors. Altogether, as also expected according to their viremia levels, patients from group A showed higher immune activation, while patients B exhibited a better immune status in PHI.

### Effect of frequency of NKG2C^+^CD57^+^ on early cART efficiency

The objective of the Optiprim trial was to evaluate the outcome of intensive versus conventional cART started during PHI on the reservoir size. A standard triple-drug regimen was compared to an intensive regimen including five drugs (darunavir, ritonavir, tenofovir disoproxil fumarate plus emtricitabine, raltegravir and maraviroc). While no differences were observed at M24 on the reservoir size, a more rapid decrease of HIV viral load was observed in patients treated with the intensive cART regimen.^23^

**Figure 5:**
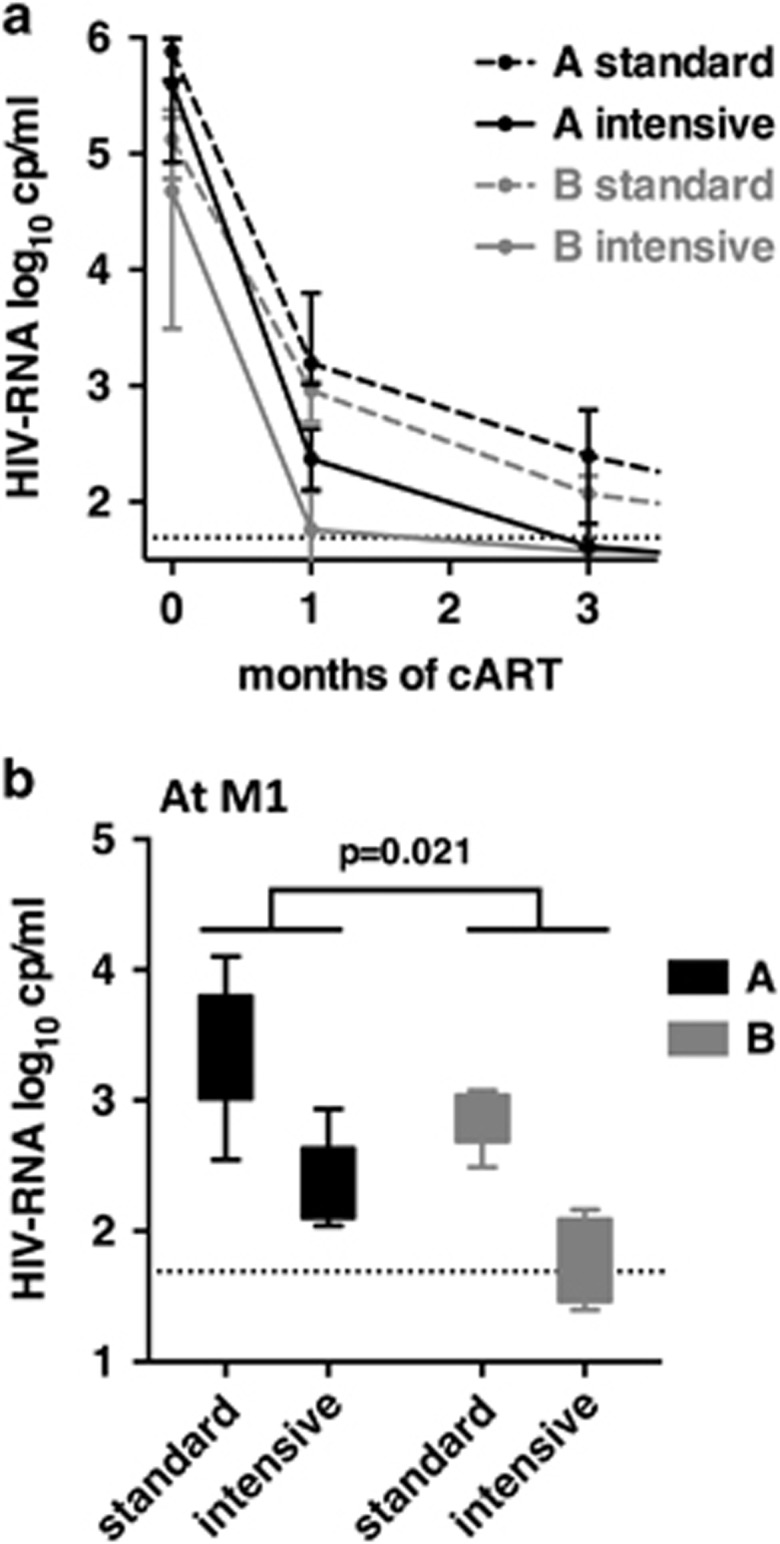
Virological response to cART at M3. (**a**) Kinetics of HIV-RNA viral load decrease in patients A and B during the 3 months of cART, according to treatment regimen (intensive and standard). Median and interquartile range are shown. (**b**) Comparison of the viral load of patients A and B at M1 according to treatment regimen. Minimum to maximum is shown. *P*-value from two-way ANOVA is shown for groups A and B. For treatment regimen, the *P*-value is <0.0001. ANOVA, analysis of variance.

The potential contribution of NKG2C^+^CD57^+^ to the early outcome of treatment was evaluated based on the kinetics of viral load decrease during the 3 months of treatment (Figure 5a). The decrease of viremia was the most rapid in group B patients treated with intensive cART and less rapid in group A patients treated with standard cART. All curves showed a two-step profile, with a rapid decrease between T0 and M1 (mean of 2.8 HIV-RNA log_10_ decrease), followed by a slower decrease between M1 and M3 (mean of 0.82 HIV-RNA log_10_ decrease). We examined thoroughly if the NKG2C^+^CD57^+^ frequency contributed to this early decrease by comparing the mean level of HIV-RNA reached by each group at M1 (Figure 5b). Patients B reached significantly lower viral load levels than patients A, regardless of the treatment regimen (*P*-value=0.021). In line with this, only patients B (two out of four) reached undetectable levels of HIV-RNA (below 50 log_10_ copies per ml) after 1 month of treatment. As shown previously, the intensive treatment showed a better efficiency than the standard one (*P*-value <0.0001) in each group of patients. Slopes of HIV-RNA decrease were similar for each group of patient treated with the same drug regimen. Accordingly, the frequency of NKG2C^+^CD57^+^ NK cells at cART onset influenced the early outcome of treatment, but the type of treatment was preponderant on the slope of HIV-RNA decrease.

## Discussion

As previously described in HIV infection,^16, 17, 18^ primary HIV-infected patients from the Optiprim-ANRS 147 study showed high frequencies of NKG2C^+^ NK cells, irrespectively of their PHI stage, stable over the first 3 months of cART, but highly variable among the patients. We went further from this observation by describing the subsets of differentiation included within the NKG2C^+^ populations, using multiparametric flow cytometry combined with bioinformatics. Distinct subsets were identified including immature CD56^bright^, effector CD56^dim^, dysfunctional CD56^neg^ and mature CD57^+^. The latter have previously been proposed as memory-like or adaptive NK cells.^8, 9^ The distribution of those phenotypes showed predominance of effector subsets in the majority of patients, while memory-like NKG2C^+^CD57^+^ NK cells were predominant in only few patients (9 out of 30). All patients studied were co-infected with CMV, proposed as the underlying factor responsible for NKG2C^+^ expansion in HIV-infected patients.^17^ The absence of active CMV reactivation suggested that HIV was the driving force responsible for the expansion of NKG2C^+^ NK cells, as previously suggested.^34^ This was supported by the positive correlation we found between NK^bright^NKG2C^+^ and HIV viral load suggesting an ongoing differentiation of NKG2C^+^ NK cells from the progenitors. Other studies also suggested that HIV,^34^ HCV^15^ and hantavirus^13^ further enhance the expansion of NKG2C^+^ cells.

While total NKG2C^+^ cells most likely expanded in response to HIV infection during PHI, it is probably not an explanation suitable for the higher levels of memory-like NKG2C^+^ NK cells found as the main NKG2C^+^ subset in the few patients of group B. NKG2C^+^CD57^+^ NK cells are found in healthy individuals with a variable magnitude. For instance, two of the unselected healthy controls included displayed, respectively, 11 and 14% of memory-like NKG2C^+^ NK cells, while the majority displayed frequencies <1%. The persistence of this NKG2C^+^ subset several months after a CMV reactivation was one reason to propose them as an adaptive or memory-like form for NK cells.^8^ In the CMV model, the memory-like CD57^+^ compartment is replenished through the maturation of effector NKG2C^+^ NK cells, several months after the acute phase of infection, once viremia is controlled.^8^ The CMV-DNA levels were however not above the reactivation threshold in the patients of this study, suggesting that CMV reactivation did not occur here during PHI. CD57^+^ NK cells have a low proliferative capacity.^25^ In our study, the individual stability of the frequencies of NKG2C^+^CD57^+^ NK cells during the 3 months of cART is consistent with this slow dynamics. Therefore, unless other unknown mechanisms contributed to expand memory-like NK cells, we speculate that the high frequencies observed in patients of group B at T0 were already high at the time of HIV infection. Conversely, the high frequencies of the effector NK^dim^NKG2C^+^ observed in the majority of patients are consistent with a rapid expansion of a subset endowed with high proliferative capacities, between infection and T0.

Individual-specific parameters linked to CMV infection might have contributed to the slightly higher levels of CMV-DNA in group B and to the higher frequencies of NKG2C^+^ memory-like NK cells in these patients. Indeed, genetic factors, such as a deletion in the *NKG2C* gene,^12^ zygosity^35^ or immune factors involved in the overall control of CMV are known to influence the frequencies of NKG2C^+^CD57^+^ NK cells in healthy carriers.

To understand the impact of the NKG2C^+^ NK cell subsets present during PHI, correlations of clinical parameters and frequencies of NKG2C^+^CD57^+^ were assessed with regard to viral load. Multivariate statistical analysis revealed that the frequency of NKG2C^+^CD57^+^ NK cells was the best parameter correlated to low HIV viral load at T0, while confounding factors such as the elapsed time between infection and enrollment, the infection stage at inclusion or the age were not correlated. Since viral load was inversely correlated with the frequencies of NKG2C^+^CD57^+^, this suggests a contribution of this NK cell subset to the natural control of infection. Immunological parameters linked to viral load or disease progression^31, 32, 33^ were consequently better in patients of group B, including higher pDC frequencies and lower immune activation. Accordingly, our results support the hypothesis that preexisting memory-like NK cells at the time of infection, possibly resulting from an individual equilibrium with CMV latent infection, constitute a partially efficient, natural response to HIV infection.

We evaluated whether high frequencies of memory-like NKG2C^+^ NK cells at the time of treatment start contributes to early response. Whereas the type of drug regimen was a preponderant factor for the rapidity of virological suppression, as previously shown for these patients,^23^ NKG2C^+^CD57^+^ NK cells seemed to contribute, albeit to a lesser extent than the drugs, to the viral load control. Patients with high frequencies of NKG2C^+^CD57^+^ NK cells at T0 reached a lower viral load after 1 month of treatment, independently of the drug regimen applied. However, the slopes of viral load decrease were similar for the two groups of patients inside each treatment type. The fact that, thanks to a contribution by the NKG2C^+^CD57^+^ NK cells, patients B always started from a lower viremia level at the onset of treatment than patients A, might have been an important parameter influencing the ability to reach undetectable viral load earlier. Altogether, high frequencies of NKG2C^+^CD57^+^ NK cells might contribute to the early cART efficiency by establishing a better environment at the time of cART onset rather than directly cooperating with treatment.

The evaluation of the capacity of patients to become post-treatment controllers after early and efficient cART followed by treatment interruption at M24 was another objective of the Optiprim study. Although it is highly speculative to correlate frequency of NKG2C^+^CD57^+^ NK cells to a multifactorial outcome, it should be mentioned that one of the two patients of the Optiprim trial who became post-treatment controllers was included in our study. This patient showed a remarkably high level of NKG2C^+^CD57^+^ at inclusion and a remarkably low level of viremia at cART onset.^23^ Recent findings suggested a major contribution of the NK compartment in the control of HIV after treatment interruption.^36^ Post-treatment-controller patients of the Visconti study demonstrated indeed a specific increase of NK cells expressing killer-cell immunoglobulin-like (KIR) receptors, stronger interferon-γ secretion when exposed to K562 cells and a higher capacity to control *in vitro* HIV infection in autologous CD4 T cells.^36^ Because of limited amount of PBMC here, functional assays were not possible. However, given the frequent co-expression of CD57 on fully mature KIR-positive NK cells,^25^ a link between the NKG2C^+^ cells described here with those highly functional NK cells is not excluded.

In summary, this study gives novel insights on NKG2C^+^ cells during early HIV infection. Memory-like NKG2C^+^ subset present at the time of infection might constitute a ready-armed immune response able to contribute to natural control of viral load, establishing a better environment for early antiviral therapy. This parameter appears to be a good candidate in the search of predictive markers to monitor HIV remission.

## Methods

### Ethics statement

The study was approved by the Sud-Mediterranee-1 Ethics Committee and the French Health Products Safety Agency, and complied with the Helsinki Declaration. All study participants provided written informed consent.

### Study population

HIV-1-infected subjects with PHI were included in a multicenter phase 3 randomized trial ANRS 147 OPTIPRIM trial (ClinicalTrials.gov, number NCT01033760). The end point was to examine at month 24 the impact on blood HIV-DNA level of intensive versus standard cART and results have been yet published.^24^ A substudy was designed to investigate parameters of innate immunity linked to cART efficacy. Among 90 patients of the main study, 30 patients were randomly included. This work shows original data on NK cells, dendritic cell (DC), CMV and plasmatic cytokines and uses information of the main study. Patient characteristics are listed in Table 1.

Fifteen healthy donor samples were obtained from the French Blood Bank (Etablissement Français du Sang) as controls. Peripheral blood mononuclear cell (PBMC) samples were frozen and kept in liquid nitrogen until tested.

### Flow cytometry

PBMC were stained with multiparametric panels containing, respectively, 9 and 12 fluorescent markers designed to investigate NK and DC populations (Supplementary Methods). Cells were incubated for 20 min at room temperature (RT) with reagents pre-mixed in phosphate buffered saline (PBS), washed, then fixed with 4% paraformaldehyde (PFA). Data were acquired on LSRII-SORP cytometer (BD Biosciences, Le Pont-de-Claix, France) equipped with four lasers (405 nm/100 mW, 488 nm/100 mW, 560 nm/50 mW and 630 nm/40 mW). Photo-multiplicator (PMT) were set using unstained and fully stained samples. Events of 7.74 × 10^6^±3.10^5^ were recorded. Compensations were performed with beads stained with corresponding reagents. Data were exported and analyzed with FlowJo (FlowJo LLC, Ashland, OR, USA) (version 9-2, MacOS X). NK cells were gated in the CD3^−^CD14^−^CD19^−^ gate as CD56^+^CD16^+^, or CD56^+^CD16^−^, or CD56^−^CD16^+^ cells. DC’s were defined as CD3^−^CD14^−^CD19^−^CD56^−^CD16^−^HLA-DR^+^ cells. For computation, a pre-analysis was performed on all samples (Supplementary Figure S1). The remaining data (3.5 × 10^5^±2.5 × 10^4^ events) were exported to create new files further subjected to automated gating (Supplementary Figure S1 and below).

### Automatic cytometry data analysis

The flowClust^37^ implementation version 3.4.11 on R version 3.1.2 (R foundation for Statistical Computing, Vienna, Austria) under Linux Cent OS 6, was applied on the following parameters: CD56-PC7, CD16-APCH7, NKG2A-PacBlue, NKG2C-PE, CD57-FITC, to compute clusters as described.^26^ The number of clusters that optimally fit the data was estimated by computing flowClust on a predefined range, and comparing them with the statistical criterion BIC and ICL (not shown). This number was estimated to be >20; therefore 27 clusters were computed in the events within the NK gate of the 60 sampled (30 patients, T0 and M3), and one healthy donor. Two samples failed to be computed. Median fluorescence intensity (MFI) values and event counts of the 1593 computed clusters were exported in an Excel table and sorted to select the 507 clusters displaying a normalized MFI value of NKG2C above or equal to 0, further analyzed in MeV.

### Multiparametric data management with MeV

MeV (https://sourceforge.net/projects/mev-tm4/)^38^ was used to visualize and group multiparametric clusters using hierarchical clustering of their centers of CD56, CD16, CD57, NKG2A and NKG2C MFI. Euclidean distance and average linkage were chosen. Prior to MeV, centers were rescaled to adjust the 5 (resp. 95) percentile of each dimension to −3 (resp. +3) and normalized. The tree was cut interactively using objective MeV tools and color interpretation of the heatmap to define populations as homogeneous groups of clusters. Names were interactively given according to the comparisons of signatures between groups, and the expression of known NK cell markers. The blocks were saved and imported in a spreadsheet program, leading to a matrix with a population identifier column associated to the initial count of events. The percentages of each population of each patient sample were summarized using pivot tables.

### Virus quantification

HIV-RNA was quantified in plasma by real-time RT-PCR with the Cobas TaqMan HIV1 v2.0 assay (Roche Diagnostics, Meylan, France). Total HIV-DNA was quantified by ultra-sensitive real-time PCR in PBMC using the Generic HIV-DNA assay from BioCentric (Bandol, France).^39^ CMV viremia was quantified by an in-house real-time PCR.^40^ Eight samples were excluded because of insufficient volumes of plasma. CMV immunoglobulins G were determined with the automated assay on LIAISON XL (DiaSorin). Results were expressed in arbitrary units.

### Innate immune activation.

#### Plasma cytokines

IP-10 concentrations were determined in stored (−80 °C) plasma samples by human Quantikine CXCL10 ELISA assay (R&D Systems, Minneapolis, MN, USA) according to the manufacturers’ instructions. Levels of IL-6 were measured on plasma frozen samples with Human IL-6 Platinum ELISA (eBioscience, Paris, France). Samples with undetectable levels of IL-6 were attributed half the minimal detectable value (0.46 pg ml^−1^).

#### Spontaneous degranulation of NK cells

PBMC samples were thawed and cultured overnight at 37 °C in RPMI medium containing 10% foetal calf serum (FCS) and IL-15 (R&D, 10 ng ml^−1^). CD107a-FITC (BD Biosciences, 1/20) was added and incubation was prolonged for 4 h. Cells were washed and stained as described using the NK cells degranulation panel.

#### DC frequencies and activation

PBMC were thawed and stained with the DC panel. Frequencies of pDC were calculated as the percentages of CD3^−^CD14^−^CD56^−^CD16^−^CD19^−^CD33^low^BDCA2^+^CD123^+^HLA-DR^+^ cells among live cells. mDC were gated as CD3^−^CD14^−^CD56^−^CD16^−^CD19^−^CD33^+^BDCA2^−^CD123^−^HLA-DR^+^ live cells, PDL-1 was quantified as a MFI ratio to isotypic control.

### Statistics

Statistical graphics were performed with Prism 6 software (GraphPad Software Inc, La Jolla CA USA). The Kruskall–Wallis test followed by multiple comparison Dunn’s post-test were used to compare variables between groups. When samples were matched, the Friedman test was used. When the number of groups was two, a simple Mann–Whitney test was reported. Correlations were evaluated by using simple linear regression analysis. Two-way ANOVA was used to compare two sets of parameters into four groups of patients.

Multivariate analyses were carried out using R 3.3.2. General linear models were computed using the glm function (Table 3). When the outcome is binary, a logistic modeling was applied, and the coefficients give the change in the log odds of the outcome for 1 unit increase in the predictor variable.

## Figures and Tables

**Table 1:** Patient characteristics at T0

	*Acute PHI*	*Early PHI*
Number of patients	14	16
Time between estimated date of infection and enrollment (days)	34 (20–53)	37 (22–55)
Standard vs intensive cART	3/11	12/4
CD4 counts at T0 (count per μl)	588 (388–1012)	518 (323–733)
CD8 counts at T0 (count per μl)	1427 (502–2716)	1946 (417–8157)
CD4 to CD8 ratio at T0	0.45 (0.21–1.05)	0.49 (0.08–1.32)
HIV-RNA at T0 (log_10_ copies per ml)	5.67 (4.6–7)	5.37 (3.2–6.8)
HIV-DNA at T0 (log_10_ copies per 10^6^ PBMC)	3.8 (3.03–4.68)	3.68 (2.78–4.48)
CD4 counts at M3 (count per μl)	758 (371–1282)	657 (424–1054)
CD8 counts at M3 (count per μl)	682 (400–1144)	625 (275–985)
HIV-RNA at M3 (log_10_ copies per ml)	1.9 (1.3–2.8)	1.84 (1.3–3.42)
HIV-DNA at M3 (log_10_ copies per 10^6^ PBMC)	3.32 (2.75–3.89)	3.31 (2.28–4.02)
Number of patients seropositive for CMV	11 (1 neg, 2 und^ed^)	15 (1 und^ed^)
CMV serology (IgG, arbitrary unit per ml)	107 (5–176)	108 (50–180)
CMV-DNA (log_10_ copies per ml)	1.66 (0.77–2.17)	2 (1–4)

Abbreviations: cART, combination antiretroviral therapy; neg:,negative; PBMC, peripheral blood mononuclear cell; PHI, primary HIV infection; unded, undetermined.

Mean values and (range) are indicated.

Acute infection was defined by one band or fewer on HIV-1 western blot and early infected by more than one band.

**Table 2:** Patient characteristics at T0 according to the groups formed on NKG2C^+^ profiles

*Characteristics*	*Groups of patients*		
	*A*	*B*	*w/o NKG2C*^*+*^
Number of patients	17	9	2
Acute vs early infection	8/9	3/6	1/1
Time between estimated date of infection and enrollment (days)	34.5 (23–55)	38 (22–46)	36 (31–41)
Standard vs intensive cART	10/7	4/5	1/1
CD4 counts (count per μl)	537 (332–1012)	593 (374–731)	390 (323–457)
CD8 counts (count per μl)	2159 (502–8157)	1081 (417–1982)	1191 (1088–1284)
CD4 to CD8 ratio	0.37 (0.08–1.3)	0.66 (0.34–1.1)	0.325
HIV-RNA (log_10_ cp per ml)	5.73 (4.6–7)	4.81 (3.2–5.5)	6.57 (6.14–7)
HIV-DNA (log_10_ cp per 10^6^ PBMC)	3.7 (3–4.7)	3.56 (2.8–4.3)	4.5 (4.48–4.52)
% of NKG2C^+^	13.2 (4.9–28.4)	7.4 (2.4–14.5)	0
% of NKG2C^+^CD57^+^	1.3 (0–5.3)	13.8 (8.6–25.5)	0
% of dysfunctional NKG2C^+^	4 (0–11.8)	2.9 (0–7.1)	19.6 (0–39.2)
CMV serology (IgG, AU ml^−1^)	99.2 (50–176)	95.3 (83–108)	136
CMV-DNA (log_10_ copies per ml)	1.71 (0.8–3.6)	1.98 (1.6–2.9)	2.32

Abbreviations: cART, combination antiretroviral therapy; PBMC, peripheral blood mononuclear cell.

T0 mean values and (range) are indicated.

**Table 3:** Multivariate statistical analysis of factors involved in HIV viral load at T0

	P*-value*	*Coef.*
PHI status (number of bands in western blot)	NS	−0.04
Time between estimated date of infection and enrollment	NS	−0.02
Age	NS	0.002
% of NKG2C^+^CD57^+^	0.0006***	−0.068

Abbreviation: NS, not significant.***, p-value<0.001
