# Supplementary material for: NKG2C+ memory-like NK cells contribute to the control of HIV viremia during primary infection: Optiprim-ANRS 147
Source: Clin Transl Immunology. 2017 Jul 7;6(7):e150–. doi: 10.1038/cti.2017.22 (PMC5539415; doi:10.1038/cti.2017.22)
Supplement: Supplementary Information [file cti201722x1.pdf]

Supplementary Information S1

Multi-parametric cytometry panels

NK panel: NKG2A-PacBlue (clone Z199, home-made), live-dead Aqua (Life Technology, 1/200), CD57-FITC (Beckman-Coulter, 1/30), NKG2C-PE (R&D, 1/40), CD14-PC5 (Beckman-Coulter, 1/30), CD19-PC5 (Beckman-Coulter, 1/30), CD56-PC7 (Beckman-Coulter; 1/30), CD3-AF700 (BD Biosciences, 1/40), CD16-APC-H7 (BD Biosciences, 1/40).

DC panel: live-dead Aqua (Life Technology, 1/200), BDCA2-FITC (Miltényi, 1/30), CD123-PercpCy5.5 (BD Biosciences, 1/20), HLA-DR-ECD (Beckmann-Coulter, 1/40), CD3-PC5 (BD Biosciences, 1/40), CD56-PC5 (Beckman Coulter, 1/30), CD19-PC5 (Beckman Coulter, 1/40), CD33-PC7 (BD Biosciences, 1/40), PDL-1-AF647 (clone 3.1, home-made coupling), CD14-APCH7 (BD Biosciences, 1/40), CD16-AF700 (BD Biosciences, 1/40). PDL-1 staining was controlled in a parallel tube containing isotypic control IgG1-AF647.

NK cells degranulation panel: CD107a-FITC (BD Biosciences, 1/20), CD56-PC7 (Beckmann-Coulter, 1/30), CD3-V450 (BD Biosciences, 1/40), live-dead Aqua (Life Technology, 1/200), and CD16-AF700 (BD Biosciences, 1/40). NK cells were gated as live CD3-CD56^{+}CD16$^{+/-}$. A control consisting of PBMC incubated without CD107 was used to set the gates for CD107 quantification.

Supplementary Figure S2

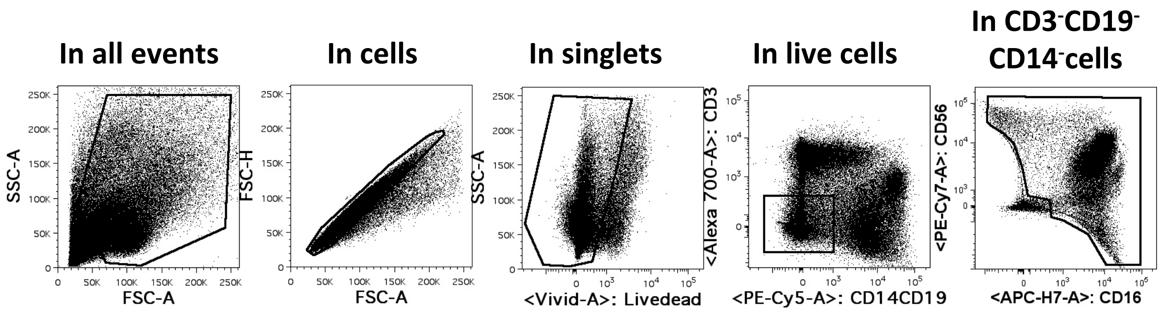

Manual gating of NK cells within patients PBMC. Left to right: exclusion of debris, doublets, dead cells and $CD3^{+}CD19^{+}CD14^{+}$ populations. NK cells are gated as $CD56^{+}CD16^{+}$, $CD56^{+}CD16^{-}$ or $CD56^{-}CD16^{+}$ cells.

Supplementary Figure S3

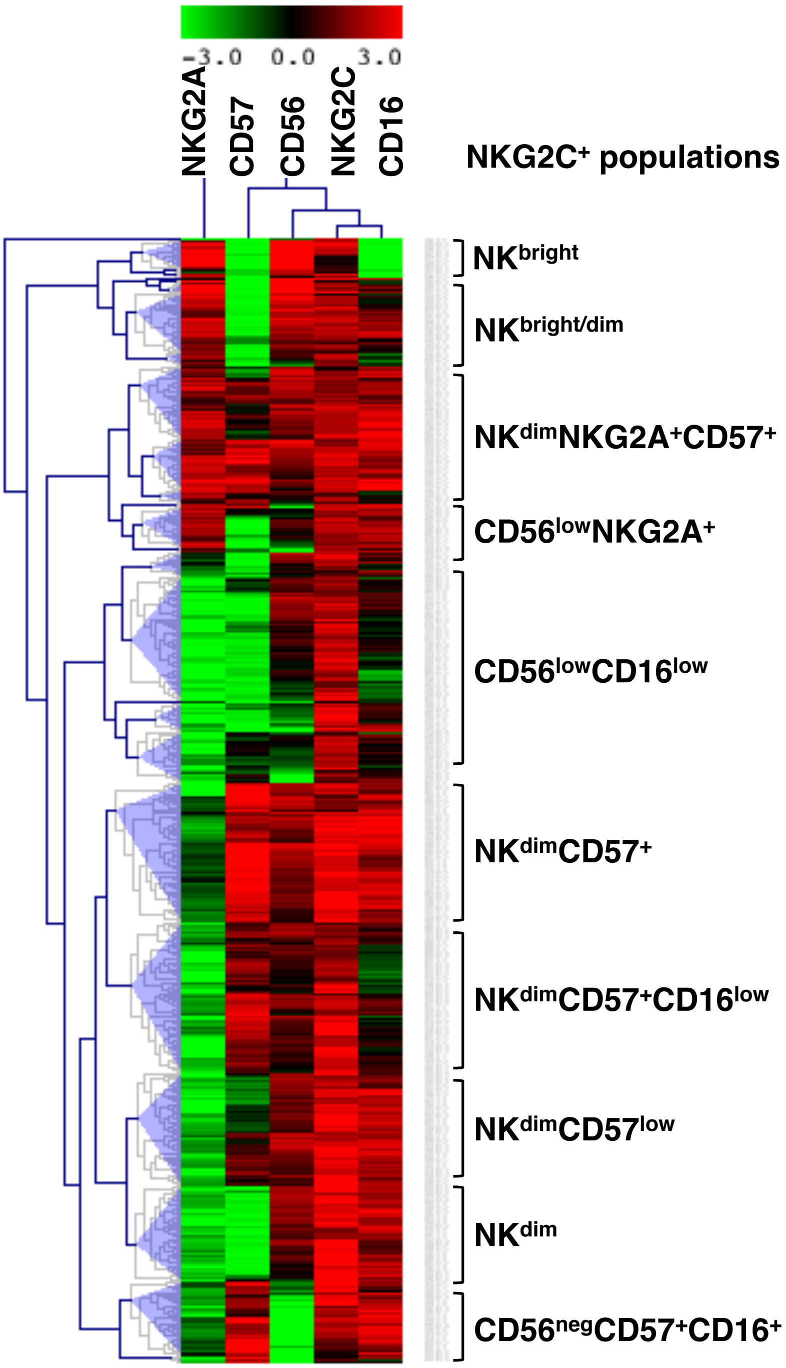

Identification of NKG2C^{+} NK cells subsets within patients samples. Visualization of the 507 NKG2C^{+} clusters computed into the NK cells files from the 30 T0 patients samples in a MeV heatmap. The clusters are visualized and merged according to their normalized MFI for the 5 markers shown on top using the MeV software HCL tools. NK populations are defined as groups of merged clusters and annotated according to their signatures shown on the right.

Supplementary Table S4

Percentages of NKG2C^{+} sub-populations within total NK cells of patients

pt id	Nkbright	Nbright/dim	NKdim2a+57+	CD56lo2a+	CD56lo16lo	NKdim57+	NKdim57+16lo	NKdim57lo	Nkdim	CD56neg57+16+
**1**	0,00	0,00	1,66	5,01	6,79	2,62	0,92	4,43	17,67	0,00
**2**	0,00	0,00	0,41	0,00	0,00	25,45	14,61	0,00	7,98	4,55
**3**	0,89	0,00	0,36	1,31	0,00	1,85	2,09	1,98	18,61	0,00
**4**	0,00	0,00	0,58	1,11	0,00	11,18	0,00	1,65	2,38	1,30
**5**	5,14	4,76	2,43	0,00	0,00	0,00	4,96	0,00	8,81	0,00
**6**	3,88	0,00	0,68	4,37	0,00	0,00	0,00	0,55	14,81	0,00
**7**	0,00	0,00	0,00	0,00	0,00	0,00	0,00	0,00	0,00	0,00
**8**	0,00	2,81	0,00	0,00	0,00	0,00	0,00	1,10	4,95	0,00
**10**	0,00	0,00	0,60	1,89	1,44	2,56	0,00	2,47	16,74	0,00
**11**	7,06	0,00	0,00	3,65	0,00	1,53	0,00	0,00	14,76	0,00
**12**	0,00	0,00	0,80	1,10	0,00	3,62	0,00	5,96	16,27	0,95
**13**	0,00	0,00	0,95	7,46	0,00	0,00	0,00	3,83	28,46	0,00
**14**	0,00	0,00	0,00	0,00	0,00	9,14	15,99	0,00	14,47	0,00
**15**	0,00	0,00	0,00	0,00	0,00	8,58	0,00	0,00	4,42	0,00
**16**	0,00	0,00	0,78	4,47	0,00	0,00	0,00	2,50	12,55	0,00
**18**	0,00	0,00	1,42	0,00	0,00	13,48	0,84	2,78	9,16	1,32
**19**	0,00	0,00	4,01	0,00	1,90	4,73	9,37	4,00	8,47	3,32
**20**	0,00	0,00	0,28	7,12	15,69	0,00	0,00	0,00	0,00	16,41
**21**	0,00	0,00	4,38	1,69	0,00	25,25	4,03	6,29	6,14	5,42
**22**	0,00	0,00	0,94	0,00	0,00	0,60	0,00	0,00	7,28	0,00
**23**	0,00	0,00	0,65	0,60	0,00	0,00	0,00	0,00	7,13	0,00
**24**	2,91	0,00	0,63	1,77	7,73	3,89	0,99	5,48	9,25	1,84
**25**	0,00	0,00	1,84	0,00	0,00	8,18	2,34	7,28	7,54	2,73
**26**	0,00	0,00	0,96	4,07	2,01	0,00	0,00	0,00	18,86	0,00
**27**	0,00	0,00	0,54	1,10	0,00	5,31	0,00	2,12	8,91	0,63
**28**	3,72	0,00	0,00	4,09	4,83	0,00	0,00	0,00	6,40	0,00
**29**	0,00	0,00	1,58	0,00	0,00	18,30	6,25	0,00	6,03	2,84
**30**	0,00	0,85	1,90	2,04	0,00	0,00	0,00	1,43	10,68	0,00

Supplementary Figure S5

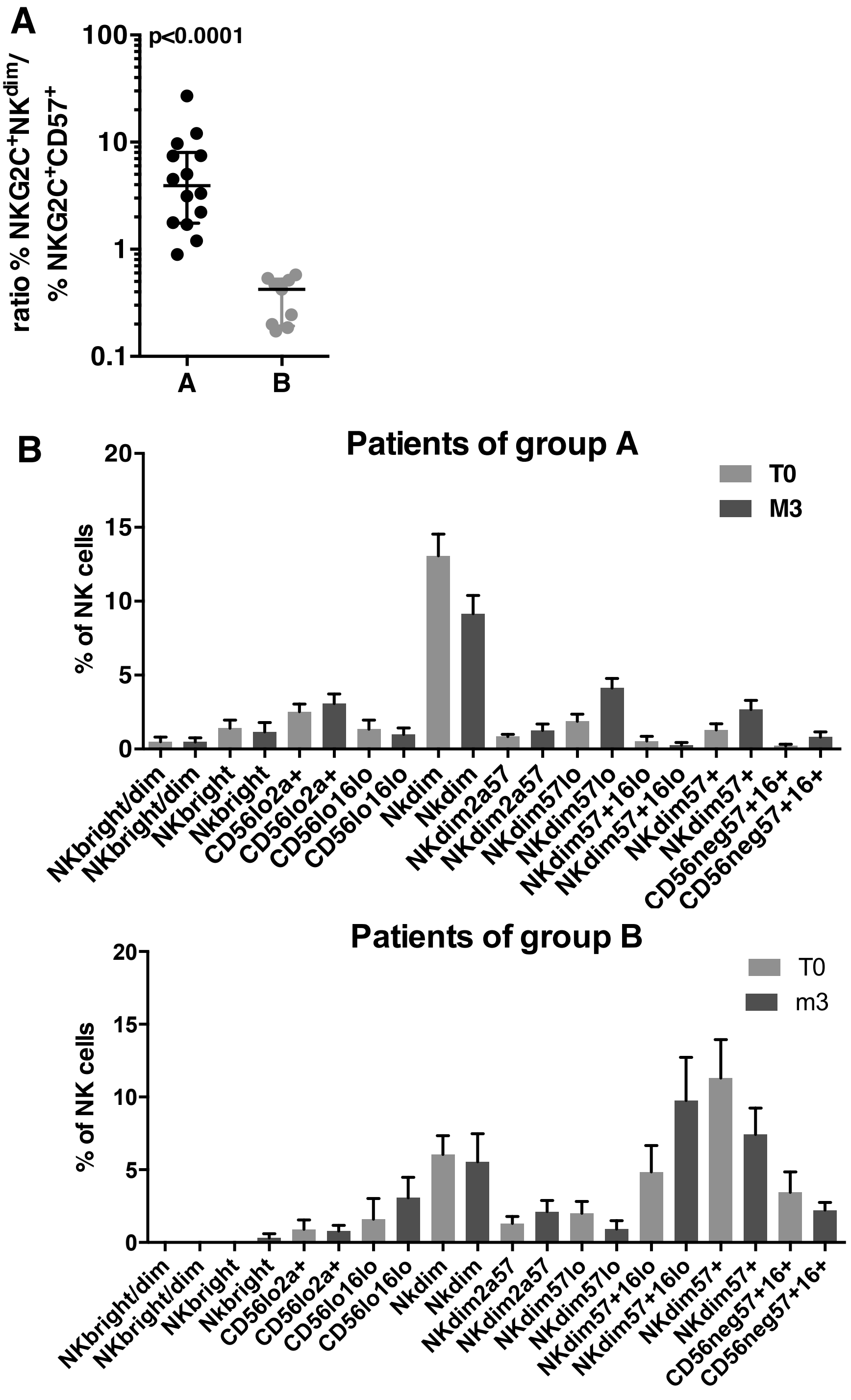

NKG2C⁺ profiles are significantly different between A and B and stable over the 3 months of cART

(A) Ratio of effector $NKG2C^{+}NK^{dim}$ to memory-like $NKG2C^{+}CD57^{+}$ in groups A and B at T0. P-value from Mann-Whitney test is indicated on top.

(B) Modifications of the percentages of each of the 10 $NKG2C^{+}$ subsets between T0 and M3 in groups A and B. None of the pairs T0-M3 are significantly different (Friedmann test.

Supplementary Figure S6

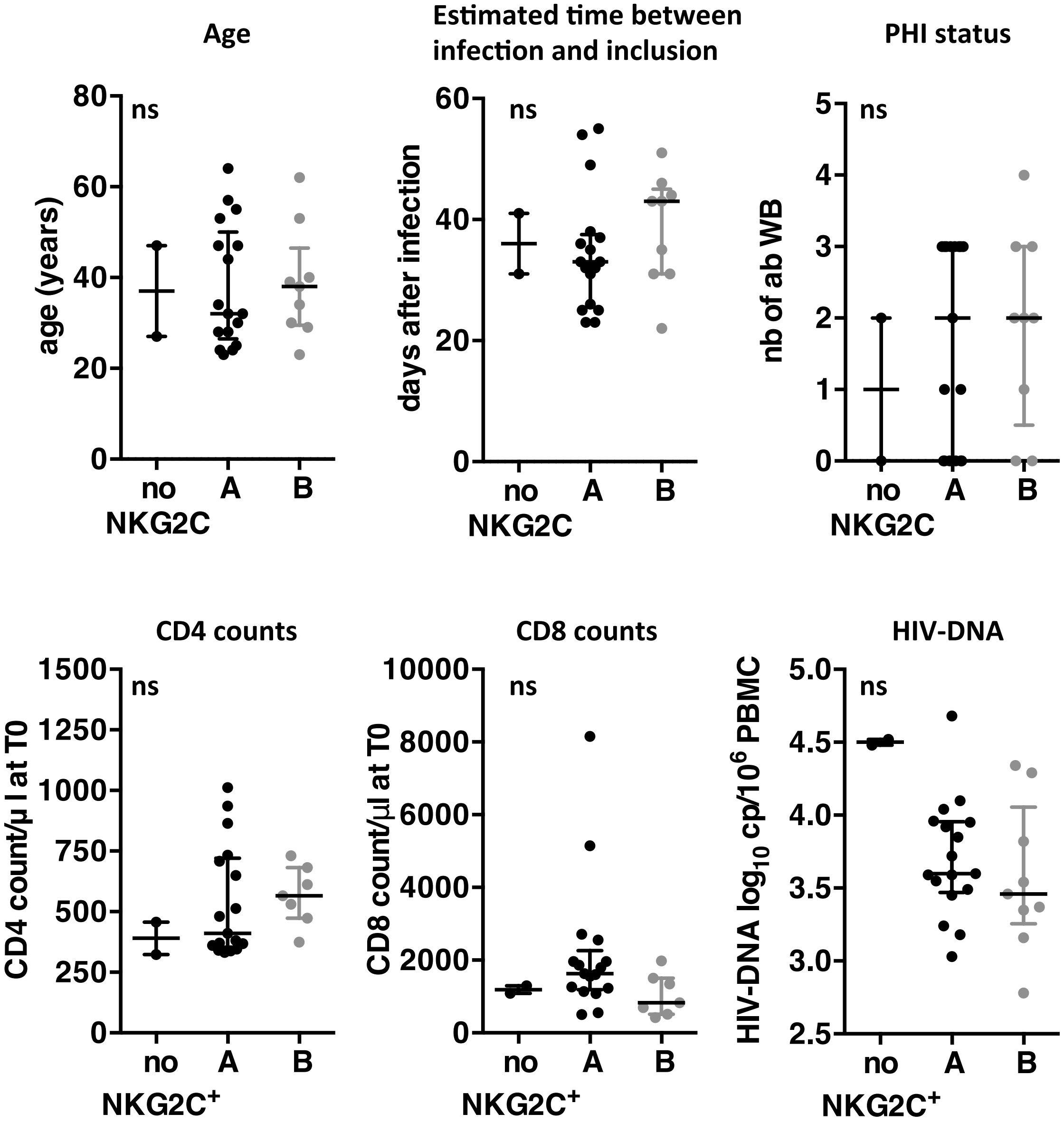

Clinical and virological parameters in patients groups at T0

Age, days after infection, PHI status estimated by the number of band on Western Blots, CD4 counts, CD8 counts and HIV-DNA in patients groups at T0. P-values from Kruskall-Wallis test is indicated on top of groups.
